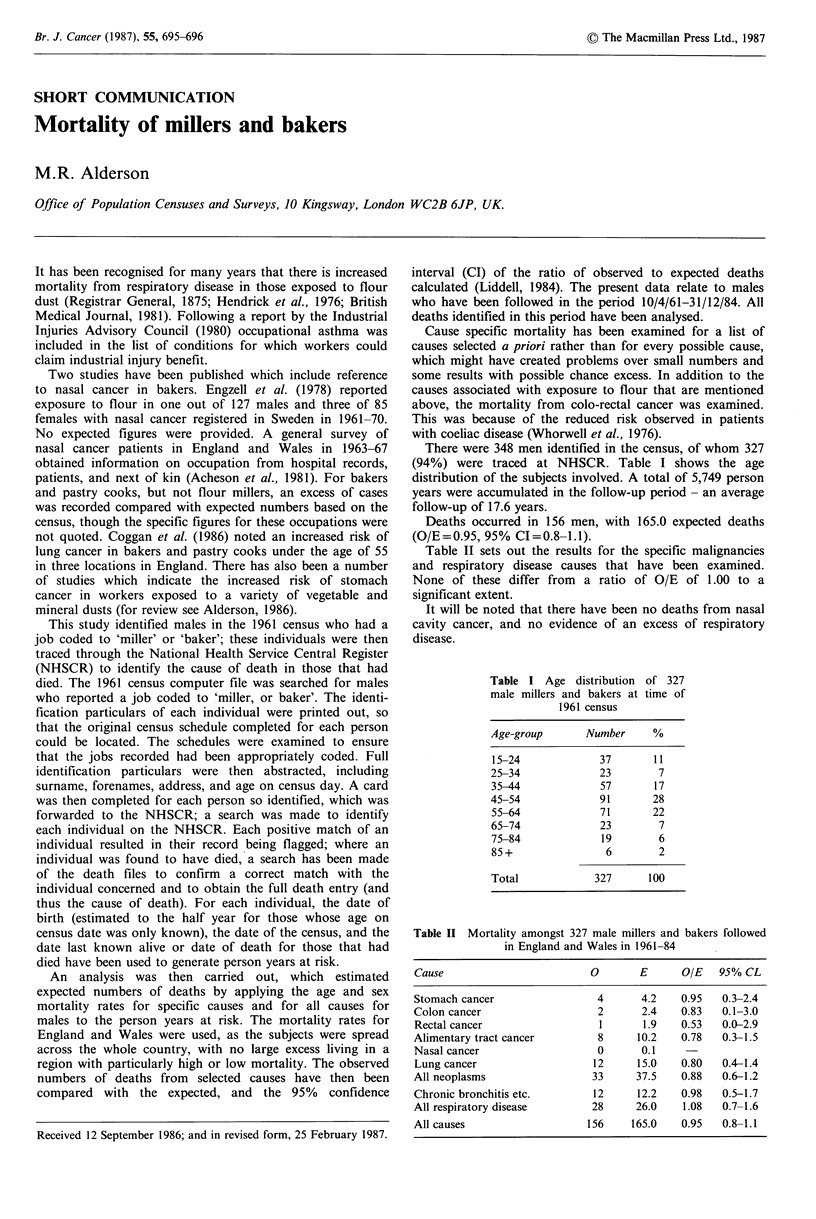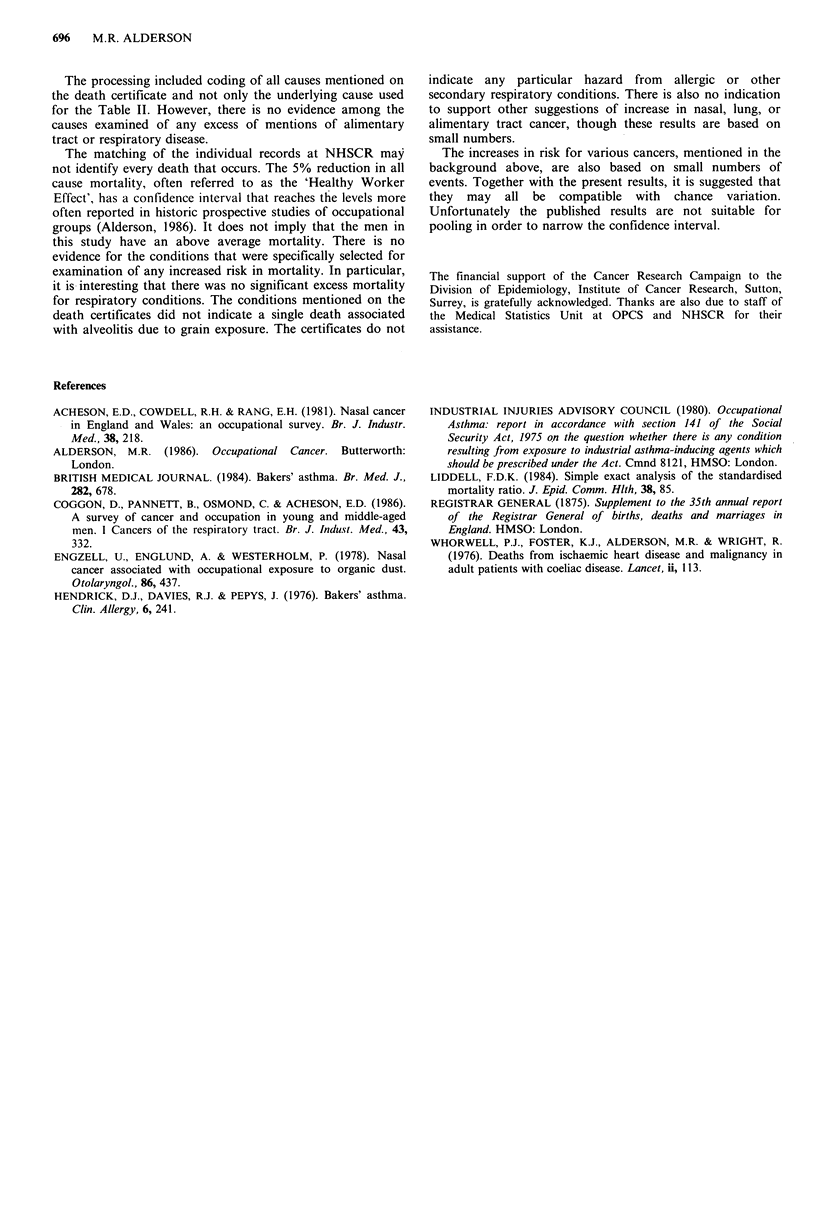# Mortality of millers and bakers.

**DOI:** 10.1038/bjc.1987.141

**Published:** 1987-06

**Authors:** M. R. Alderson


Br. J. Cancer (1987), 55, 695–696 © The Macmillan Press Ltd., 1987

SHORT COMMUNICATION

### Mortality of millers and bakers

M.R. Alderson

*Office of Population Censuses and Surveys, 10 Kingsway, London WC2B 6JP, UK.*

It has been recognised for many years that there is increased mortality from respiratory disease in those exposed to flour dust (Registrar General, 1875; Hendrick *et al.*, 1976; British Medical Journal, 1981). Following a report by the Industrial Injuries Advisory Council (1980) occupational asthma was included in the list of conditions for which workers could claim industrial injury benefit.

Two studies have been published which include reference to nasal cancer in bakers. Engzell *et al.* (1978) reported exposure to flour in one out of 127 males and three of 85 females with nasal cancer registered in Sweden in 1961–70. No expected figures were provided. A general survey of nasal cancer patients in England and Wales in 1963–67 obtained information on occupation from hospital records, patients, and next of kin (Acheson *et al.*, 1981). For bakers and pastry cooks, but not flour millers, an excess of cases was recorded compared with expected numbers based on the census, though the specific figures for these occupations were not quoted. Coggan *et al.* (1986) noted an increased risk of lung cancer in bakers and pastry cooks under the age of 55 in three locations in England. There has also been a number of studies which indicate the increased risk of stomach cancer in workers exposed to a variety of vegetable and mineral dusts (for review see Alderson, 1986).

This study identified males in the 1961 census who had a job coded to 'miller' or 'baker'; these individuals were then traced through the National Health Service Central Register (NHSCR) to identify the cause of death in those that had died. The 1961 census computer file was searched for males who reported a job coded to 'miller, or baker'. The identification particulars of each individual were printed out, so that the original census schedule completed for each person could be located. The schedules were examined to ensure that the jobs recorded had been appropriately coded. Full identification particulars were then abstracted, including surname, forenames, address, and age on census day. A card was then completed for each person so identified, which was forwarded to the NHSCR; a search was made to identify each individual on the NHSCR. Each positive match of an individual resulted in their record being flagged; where an individual was found to have died, a search has been made of the death files to confirm a correct match with the individual concerned and to obtain the full death entry (and thus the cause of death). For each individual, the date of birth (estimated to the half year for those whose age on census date was only known), the date of the census, and the date last known alive or date of death for those that had died have been used to generate person years at risk.

An analysis was then carried out, which estimated expected numbers of deaths by applying the age and sex mortality rates for specific causes and for all causes for males to the person years at risk. The mortality rates for England and Wales were used, as the subjects were spread across the whole country, with no large excess living in a region with particularly high or low mortality. The observed numbers of deaths from selected causes have then been compared with the expected, and the 95% confidence interval (CI) of the ratio of observed to expected deaths calculated (Liddell, 1984). The present data relate to males who have been followed in the period 10/4/61–31/12/84. All deaths identified in this period have been analysed.

Cause specific mortality has been examined for a list of causes selected *a priori* rather than for every possible cause, which might have created problems over small numbers and some results with possible chance excess. In addition to the causes associated with exposure to flour that are mentioned above, the mortality from colo-rectal cancer was examined. This was because of the reduced risk observed in patients with coeliac disease (Whorwell *et al.*, 1976).

There were 348 men identified in the census, of whom 327 (94%) were traced at NHSCR. Table I shows the age distribution of the subjects involved. A total of 5,749 person years were accumulated in the follow-up period – an average follow-up of 17.6 years.

Deaths occurred in 156 men, with 165.0 expected deaths (O/E=0.95, 95% CI=0.8–1.1).

Table II sets out the results for the specific malignancies and respiratory disease causes that have been examined. None of these differ from a ratio of O/E of 1.00 to a significant extent.

It will be noted that there have been no deaths from nasal cavity cancer, and no evidence of an excess of respiratory disease.

**Table I** Age distribution of 327 male millers and bakers at time of 1961 census

| *Age-group* | *Number* | % |
|---|---|---|
| 15–24 | 37 | 11 |
| 25–34 | 23 | 7 |
| 35–44 | 57 | 17 |
| 45–54 | 91 | 28 |
| 55–64 | 71 | 22 |
| 65–74 | 23 | 7 |
| 75–84 | 19 | 6 |
| 85+ | 6 | 2 |
| Total | 327 | 100 |

**Table II** Mortality amongst 327 male millers and bakers followed in England and Wales in 1961–84

| *Cause* | *O* | *E* | *O/E* | *95% CL* |
|---|---|---|---|---|
| Stomach cancer | 4 | 4.2 | 0.95 | 0.3–2.4 |
| Colon cancer | 2 | 2.4 | 0.83 | 0.1–3.0 |
| Rectal cancer | 1 | 1.9 | 0.53 | 0.0–2.9 |
| Alimentary tract cancer | 8 | 10.2 | 0.78 | 0.3–1.5 |
| Nasal cancer | 0 | 0.1 | — | |
| Lung cancer | 12 | 15.0 | 0.80 | 0.4–1.4 |
| All neoplasms | 33 | 37.5 | 0.88 | 0.6–1.2 |
| Chronic bronchitis etc. | 12 | 12.2 | 0.98 | 0.5–1.7 |
| All respiratory disease | 28 | 26.0 | 1.08 | 0.7–1.6 |
| All causes | 156 | 165.0 | 0.95 | 0.8–1.1 |

Received 12 September 1986; and in revised form, 25 February 1987.

The processing included coding of all causes mentioned on the death certificate and not only the underlying cause used for the Table II. However, there is no evidence among the causes examined of any excess of mentions of alimentary tract or respiratory disease.

The matching of the individual records at NHSCR may not identify every death that occurs. The 5% reduction in all cause mortality, often referred to as the 'Healthy Worker Effect', has a confidence interval that reaches the levels more often reported in historic prospective studies of occupational groups (Alderson, 1986). It does not imply that the men in this study have an above average mortality. There is no evidence for the conditions that were specifically selected for examination of any increased risk in mortality. In particular, it is interesting that there was no significant excess mortality for respiratory conditions. The conditions mentioned on the death certificates did not indicate a single death associated with alveolitis due to grain exposure. The certificates do not indicate any particular hazard from allergic or other secondary respiratory conditions. There is also no indication to support other suggestions of increase in nasal, lung, or alimentary tract cancer, though these results are based on small numbers.

The increases in risk for various cancers, mentioned in the background above, are also based on small numbers of events. Together with the present results, it is suggested that they may all be compatible with chance variation. Unfortunately the published results are not suitable for pooling in order to narrow the confidence interval.

The financial support of the Cancer Research Campaign to the Division of Epidemiology, Institute of Cancer Research, Sutton, Surrey, is gratefully acknowledged. Thanks are also due to staff of the Medical Statistics Unit at OPCS and NHSCR for their assistance.

**References**

ACHESON, E.D., COWDELL, R.H. & RANG, E.H. (1981). Nasal cancer in England and Wales: an occupational survey. *Br. J. Industr. Med.*, **38,** 218.

ALDERSON, M.R. (1986). *Occupational Cancer.* Butterworth: London.

BRITISH MEDICAL JOURNAL. (1984). Bakers' asthma. *Br. Med. J.*, **282,** 678.

COGGON, D., PANNETT, B., OSMOND, C. & ACHESON, E.D. (1986). A survey of cancer and occupation in young and middle-aged men. I Cancers of the respiratory tract. *Br. J. Indust. Med.*, **43,** 332.

ENGZELL, U., ENGLUND, A. & WESTERHOLM, P. (1978). Nasal cancer associated with occupational exposure to organic dust. *Otolaryngol.*, **86,** 437.

HENDRICK, D.J., DAVIES, R.J. & PEPYS, J. (1976). Bakers' asthma. *Clin. Allergy,* **6,** 241.

INDUSTRIAL INJURIES ADVISORY COUNCIL (1980). *Occupational Asthma: report in accordance with section 141 of the Social Security Act, 1975 on the question whether there is any condition resulting from exposure to industrial asthma-inducing agents which should be prescribed under the Act.* Cmnd 8121, HMSO: London.

LIDDELL, F.D.K. (1984). Simple exact analysis of the standardised mortality ratio. *J. Epid. Comm. Hlth,* **38,** 85.

REGISTRAR GENERAL (1875). *Supplement to the 35th annual report of the Registrar General of births, deaths and marriages in England.* HMSO: London.

WHORWELL, P.J., FOSTER, K.J., ALDERSON, M.R. & WRIGHT, R. (1976). Deaths from ischaemic heart disease and malignancy in adult patients with coeliac disease. *Lancet,* **ii,** 113.